# Different Stimuli, Different Spatial Codes: A Visual Map and an Auditory Rate Code for Oculomotor Space in the Primate Superior Colliculus

**DOI:** 10.1371/journal.pone.0085017

**Published:** 2014-01-15

**Authors:** Jungah Lee, Jennifer M. Groh

**Affiliations:** Center for Cognitive Neuroscience, Department of Psychology and Neuroscience, Department of Neurobiology, Duke University, Durham, North Carolina, United States of America; University College London, United Kingdom

## Abstract

Maps are a mainstay of visual, somatosensory, and motor coding in many species. However, auditory maps of space have not been reported in the primate brain. Instead, recent studies have suggested that sound location may be encoded via broadly responsive neurons whose firing rates vary roughly proportionately with sound azimuth. Within frontal space, maps and such rate codes involve different response patterns at the level of individual neurons. Maps consist of neurons exhibiting circumscribed receptive fields, whereas rate codes involve open-ended response patterns that peak in the periphery. This coding format discrepancy therefore poses a potential problem for brain regions responsible for representing both visual and auditory information. Here, we investigated the coding of auditory space in the primate superior colliculus(SC), a structure known to contain visual and oculomotor maps for guiding saccades. We report that, for visual stimuli, neurons showed circumscribed receptive fields consistent with a map, but for auditory stimuli, they had open-ended response patterns consistent with a rate or level-of-activity code for location. The discrepant response patterns were not segregated into different neural populations but occurred in the same neurons. We show that a read-out algorithm in which the site and level of SC activity both contribute to the computation of stimulus location is successful at evaluating the discrepant visual and auditory codes, and can account for subtle but systematic differences in the accuracy of auditory compared to visual saccades. This suggests that a given population of neurons can use different codes to support appropriate multimodal behavior.

## Introduction

The superior colliculus is an important model system for the integration of spatial information from different sensory modalities [1]. Previous studies have suggested that the SC contains congruent maps of visual and auditory space (ferret: [2], [3]; guinea pig: [4]; barn owl: [5] ; cat: [6]–[8]; bats: [9]–[11]).

**Figure 1:**
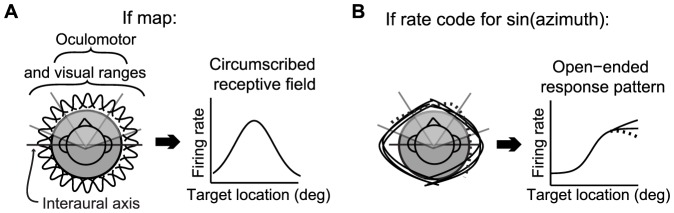
Different spatial coding formats. When sampling of space must be limited to the oculomotor or visual ranges, maps and rate codes for sound azimuth can be distinguished by evaluating whether neurons exhibit circumscribed receptive fields (A) or open-ended response functions (B). Rate coding neurons might show some degree of non-monotonicity if their underlying tuning functions were not all perfectly aligned with the interaural axis (dotted line).

The aligned-map hypothesis of multisensory integration presupposes that auditory space is indeed encoded via a map. Such a map should contain neurons whose receptive fields tile the entire expanse of space (Figure 1A). When tested with stimuli limited to the oculomotor or visual ranges - the ranges of space relevant for the rostral, eye movement-related, superior colliculus - the signature feature that best characterizes maps is that such receptive fields would appear *circumscribed*. That is, a sizeable population of neurons should have peak responses at some best target location within the tested range, and much lower responses for targets on either side. Sound location would be reflected in the identity of the active population.

However, other ways of encoding stimulus position are known to exist, particularly for auditory information in primates. In monkeys and humans, auditory-responsive neurons in areas upstream from the SC do not appear to have such bounded receptive fields distributed across the scene [12]–[17]. Instead, their “receptive fields” exhibit an open-ended structure in frontal space with peak activity in the periphery. These open-ended response patterns likely derive from interaural timing and/or level differences, which reach their maximum values for sounds located along the interaural axis (Figure 1B, peaks of “receptive fields” cluster at left and right poles). Sound location would then be encoded not by the identity of the active population but by the level of neural activity, being proportional to the sine of the azimuthal location of the stimulus. Such a code is referred to as a rate code for sound location.

In this study, we investigated the coding format of auditory responses of rostral SC neurons in detail, to ascertain whether they exhibit a clos­ed-field organization, similar to visually-driven activity in this structure and consistent with the formation of a map of space, or whether they are open-field, like the response patterns of neurons in the auditory areas that serve as inputs to the SC, and therefore potentially consistent with a rate code for sound location.

## Results

### Behavioral paradigm and analysis of neural data

**Figure 2:**
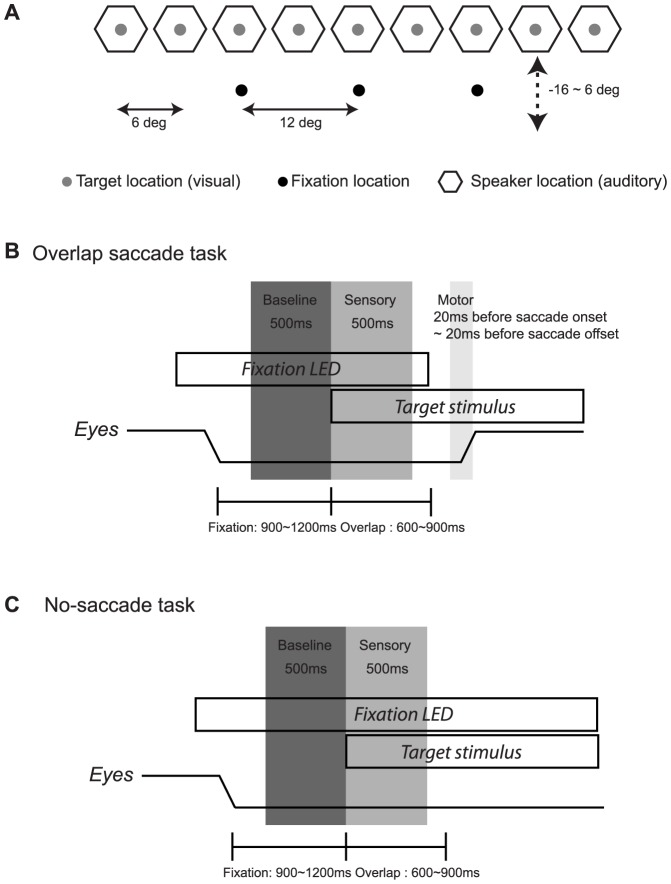
Experimental design. Spatial layout of the targets (A) shows that the fixation targets (black dots) were located 12° left, 0° and 12° right at varying elevations depending on the spatial sensitivity of the neuron under study ranged −16 to 6 degree (mean±SD: −4.2±4,1). Targets were either auditory (white noise burst) or visual (LED), presented from a stimulus array of 9 speakers each with an LED attached to its face. The speakers were spaced from 24° left to 24° right with 6° intervals at an elevation of 0° with respect to the animal’s head. B. Events of the overlap saccade task. The baseline period was 500 ms before target onset, the sensory period was 0−500 ms after target onset, and the motor period began 20 ms before saccade onset and ended 20 ms before saccade offset. C. The no-saccade task was similar except that the targets were near or beyond the oculomotor range, and the animal was not required to make an eye movement because the fixation light stayed on.

We assessed the responses of SC neurons (n = 180) in monkeys making eye movements to visual and auditory targets. Target locations spanned a range of +/− 24° with respect to the head from three initial fixation positions (−12, 0, 12°, for a range of +/− 36° with respect to the eyes.) Monkeys (n = 2) performed an overlap saccade task (Figure 2; see also Materials and Methods) allowing sensory-related activity (0−500 ms after the target) to be dissociated from saccade-related activity (20 ms before saccade onset to 20 ms before saccade offset). We selected neurons that responded significantly for at least one target modality (n = 175, 97% out of 180 neurons, Table 1) for further study. The majority were bimodal (63 and 84% for sensory and saccade-related activity respectively). The response patterns for visual stimuli were predominantly eye-centered during both the sensory and motor periods, whereas auditory activity shifted from hybrid to eye-centered coordinates as the saccade approached. This and other aspects of this neural data set are described in more detail in our previous study [18]. To eliminate reference frame as a factor for the present study, we conducted all analyses separately for each fixation position.

**Table 1:** Neural responses were tested for statistical significance during sensory and motor periods in comparison to the baseline period.

Overlap saccade task (N = 180)	Visual		Auditory		Both	
	N	Total (%)	N	Total (%)	N	Total (%)
A) sensory response (two sample t-test p<0.05)	159	88.3	122	67.8	113	62.8
B) motor response (two sample t-test p<0.05)	162	90.0	159	88.3	151	83.9
C) A and B	155	86.1	117	65.0	111	61.7
No-saccade task (N = 148)						
sensory response (two sample t-test p<0.05)	142	96.0	111	75.0	105	70.9

The time periods in relation to the events of the task are illustrated in Figure 2. Neurons were included for subsequent analyses on the basis of these screening tests.

### Differences between visual and auditory spatial coding

**Figure 3:**
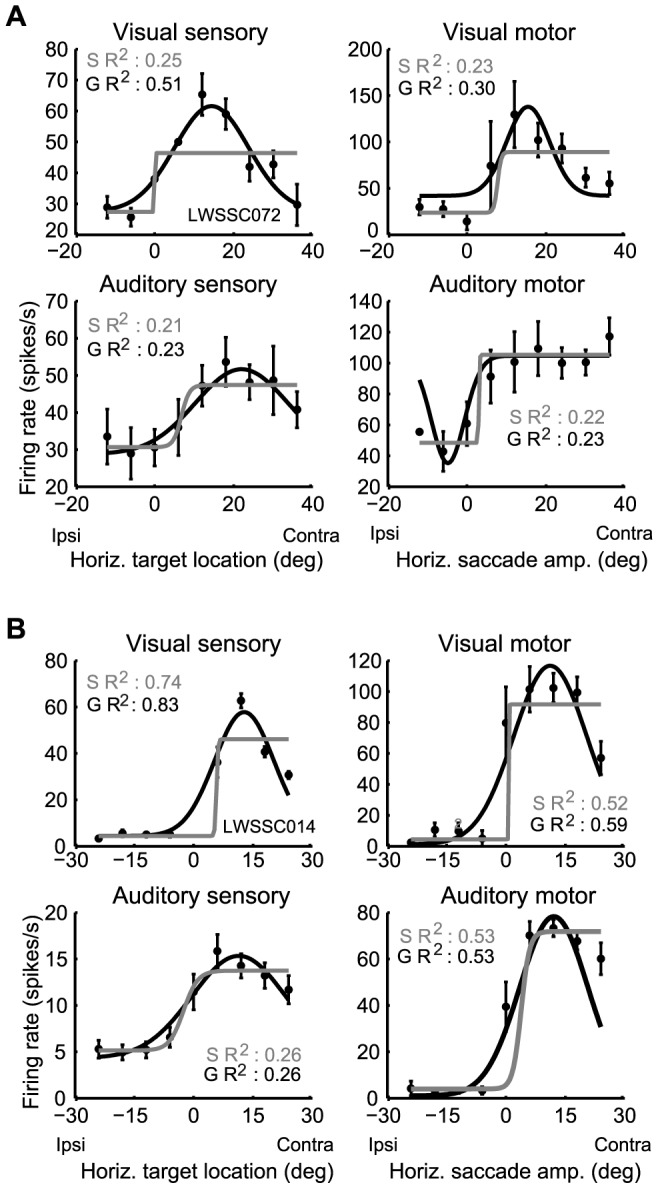
Two representative SC neurons (A, B) showing different sensitivity for visual and auditory stimuli (mean discharge rate +/− standard error with respect to the horizontal eye-centered target location or movement amplitude; S R^2^ and G R^2^ refer to the Sigmoidal and Gaussian R^2^ values). For three out of the four visual responses (upper panels), the fits of Gaussian function are significantly better than those of sigmoidal function (the sensory R^2^ values for A and B, and the motor R^2^ value for B; bootstrap analysis, p<0.05). In contrast, for the auditory responses (lower panels), the fit of both functions are about equally good (bootstrap analysis, p > 0.05).

Neurons showed different spatial response properties depending on whether the target was visual or auditory. For visual stimuli, the classic circumscribed receptive field pattern was evident: responses were largest for a particular target eccentricity, but fell off substantially for targets located both more centrally and more peripherally. For example, the neuron shown in Figure 3A shows a peak response for visual targets/saccades at about 18 degrees contralateral, for both the sensory and motor period. Activity for both larger and smaller amplitude target displacements (e.g. 0 or 40°) is considerably lower. The visual responses of the neuron in Figure 3B are similar.

In contrast, for auditory stimuli, responses typically showed an open-ended pattern. As target eccentricity increased, responses either continued to increase, reached a plateau, or showed only a modest dip in activity. The auditory motor responses of the neuron in Figure 3A (bottom right panel) reached a plateau at around 15° and did not drop from this level. The auditory responses of the neuron in 3B show a small decrease in activity for larger amplitude targets (Note that the visual and auditory responses within 3A are from the same neuron, as are those of 3B for a different neuron).

**Figure 4:**
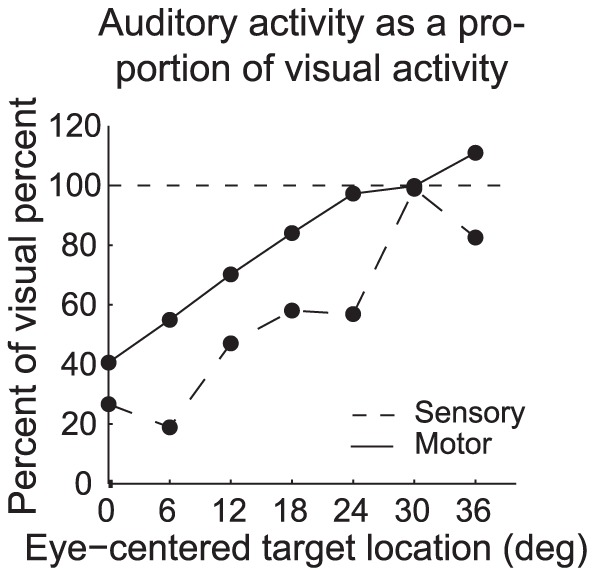
“Point image” of auditory activity in comparison to visual activity as a function of target location. For each neuron, we calculated the activity for a given target location, modality, or response period as a proportion of the peak firing rate observed for any target location, modality, or response period for that neuron. We then calculated the average of this normalized activity across the population of neurons as a function of target modality and target location. This graph plots the average normalized population activity on auditory trials as a percentage of that observed on visual trials. (Only locations in the contralateral hemisphere are shown because visual activity is very low or non-existent for ipsilateral targets, which would make even modest auditory activity appear very large in comparison.) A value of 100 (horizontal dotted line) indicates that the activity for visual and auditory stimuli at the corresponding target location was about equal. As target location becomes more eccentric, the level of activity evoked by auditory stimuli during the motor period approaches and then slightly exceeds that observed for visual stimuli (solid line). A similar increase in auditory activity relative to visual activity with target eccentricity is observed during the sensory period (dashed line), but at an overall lower level.

A difference is also evident in the “point image” of activity evoked on visual vs auditory trials. Figure 4 shows the average activity on auditory trials normalized to that observed for visual trials as a function of target location within the contralateral hemifield. The relative activity levels increase with increasing target eccentricity, both during the sensory period and during the motor period, For the most eccentric targets (>30°), auditory motor-related activity could exceed that observed for visual targets (y values greater than 100).

### Quantitative population analysis

To quantitatively measure this difference across the population, we reasoned that a circumscribed receptive field should be fit *better* by a Gaussian than a sigmoidal function. In contrast, an open-ended response pattern with its generally monotonic shape should be fit by *either* a sigmoid or a Gaussian – given that the left or right half of a Gaussian is quite similar to a sigmoid [12]. For the example neurons shown in Figure 3, the coefficients of determination, R^2^, of the Gaussian and sigmoidal fits are both significant for visual responses. However, the Gaussian R^2^ is larger, and a bootstrap analysis shows that this difference is significant for the visual sensory examples and one of the two visual motor examples (the sigmoidal R^2^ was calculated for 100 iterations with 80% of data for each target location; if the full-data Gaussian R^2^ exceeded 95% of these subsampled sigmoidal R^2^ values, it was classed as significantly greater than the sigmoidal R^2^ at p<0.05). In contrast, for the auditory responses of the same neurons the Gaussian and the sigmoid curve capture about an equal amount of the variance, indicating that the relationship between activity and sound location is statistically indistinguishable from being open-ended (bootstrap analysis, p >0.05).

**Figure 5:**
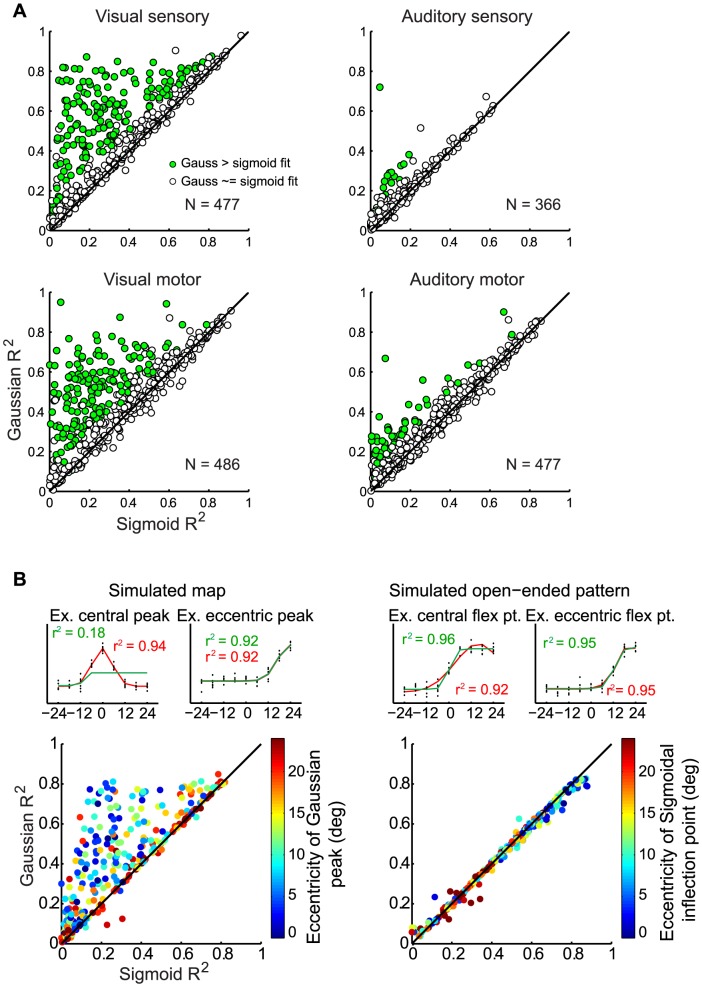
Comparison of the goodness of fit for Gaussian versus sigmoidal functions. Results for the population of SC neurons (A), with the color and symbol type indicating whether the Gaussian curve fit was significantly superior to that of the sigmoid (bootstrap analysis, p<0.05). Each neuron contributed 3 points to these panels, one for each fixation position. B. Simulation of the expected R^2^ values of Gaussian and sigmoidal curves if the underlying functions are Gaussian (left) vs sigmoidal (right). Units were simulated as Gaussians or sigmoids of varying parameters with noise, then fits were calculated for each unit and plotted in color indicating the location of the peak (left panel) or inflection point (right panel). The examples illustrate individual units with different peak or inflection point locations. (See: Table 2 for statistical significance of fits; Figure S1 for the experimental data limited to bimodal neurons; Figure S2 for the real data color coded by the eccentricity of the peak of the Gaussian or inflection point of the sigmoid; Figure S3 for data at sites tested with microstimulation; Figure S4 for the same data plotted as a correlation coefficient R for comparison with our previous studies [12], [15]; and Figure S5 for histograms of the difference between the Gaussian and sigmoidal R^2^; Figure S6 for the subset matching the visual and auditory responsiveness; and Figure S7 for the subset matching the visual and auditory response variability)

At the level of individual response patterns, the differences between visual and auditory spatial sensitivity are small, but at the population level they are consistent. Figure 5A compares the Gaussian vs. sigmoidal R^2^ *s*, depending on whether the target was visual or auditory, for all the responsive neurons. For visual targets, the R^2^ of the Gaussian fits is frequently larger than that of the sigmoidal fit (data points above the line of slope one), consistent with circumscribed receptive fields. This is true of both the sensory period and the motor period (green dots in left panels in Figure 5A; 34.6% for sensory, 32.1% for motor, Table 2). In contrast, for auditory targets, the Gaussian fits tend not to be much better than the sigmoidal fits (green dots in right panels in Figure 5A; 4.4% for sensory, 10.8% for motor, Table 2). The data lie along the diagonal, indicating open-ended response patterns across the population. The same was true for the subset of the population that was bimodal for the response period in question, i.e. the visual and auditory spatial sensitivity patterns of exactly the same neurons (Figure S1). The pattern remained when modality-dependent differences in overall responsiveness or variability were eliminated (Figure S6-7).

**Table 2:** Summary of the statistical significance of the curve fits shown in Figure 5A.

	N	Gaussian and Sigmoid (p<0.05, %)	Gaussian only	Sigmoid only	Neither	Bootstrap Gaussian > Sigmoid (p<0.05,%)
Visual sensory	477	83.6	10.5	0.0	5.9	34.6
Visual motor	486	87.4	8.8	0.0	3.7	32.1
Auditory sensory	366	54.9	10.1	1.1	33.9	4.4
Auditory motor	477	83.4	9.2	0.4	6.9	10.7

Column 1: Each included neuron contributed three fits, one for each eye position. Columns 2−5: The proportion of Gaussian and sigmoidal curve fits that were individually significant. Column 6: The proportion of neuron-eye position combinations for which the observed Gaussian R^2^ value was significantly greater than 95% of the bootstrapped sigmoidal R^2^ values generated for that neuron and eye position; corresponds to the proportion of green data points in Figure 5A.

### Comparison to simulated data

To verify that this comparison between Gaussian and sigmoidal fitting can successfully distinguish between such response patterns, we tested the curve fitting procedure on simulated Gaussian and sigmoidal data plus noise (see Materials and Methods). For open-ended response patterns simulated with sigmoids, the sigmoidal and Gaussian curve fits were equally successful (the R^2^ values are essentially identical and the data lie along the line of slope one, Figure 5B right panel) and the resulting curves are indistinguishable from each other in shape (examples). For circumscribed receptive fields simulated with Gaussians, the Gaussian fits tended to yield higher R^2^ values than did the sigmoidal fits (data points largely above the line of slope one, Figure 5B left panel). The relative advantage of a Gaussian fit depended on the eccentricity of the peak location with respect to the sampled range. For simulated units tuned to the center location, the Gaussian provided a much better fit (see examples). In contrast, if a simulated neuron’s peak tuning was more peripheral with respect to the sampled range, the sigmoidal function also yielded a good fit.

### Controlling for the sampling range

Could the apparent open-ended auditory response patterns in the actual neurons therefore be an artifact of failing to sample circumscribed auditory receptive fields at sufficiently eccentric locations? Several points argue against this interpretation. First, the visual and auditory targets occupied the same locations, so the sampling of visual and auditory space was identical. If the sampling was insufficient to observe circumscribed auditory receptive fields, it should also have been insufficient for visual receptive fields. Second, the sampling was matched to the range of space where circumscribed receptive fields should have been found if they existed. The targets spanned the portion of the oculomotor range of monkeys that is not normally accompanied by head movements [19]. Furthermore, we concentrated on sampling the rostral region of the SC which codes for smaller saccades: the mean stimulation-evoked horizontal component of saccade amplitude at 19 representative sites was 6.4°, and the range was from 0.67 to 15.2° horizontally (see Materials and Methods)(Figure S3). Thus our sampling, out to 36° in eye-centered coordinates generally extended beyond the expected range of peak movement field locations in our neural data.

**Figure 6:**
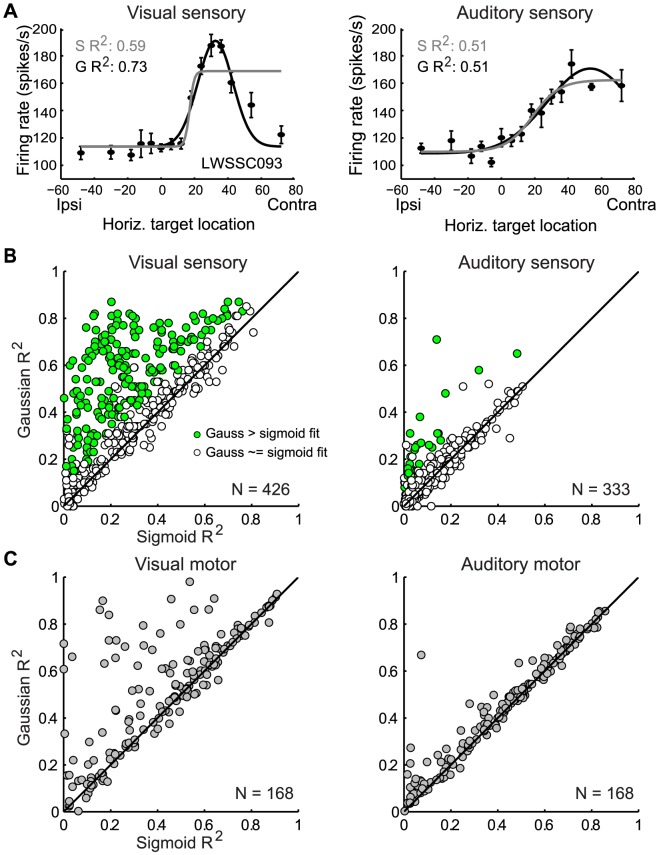
Two tests of the effect of sampling range. Results in for an example neuron (A) tested out to 72° relative to the eye (ipsilateral fixation, interleaved non-saccade task). B. Population results, format similar to the corresponding panels of Figure 5A. C. Results for the motor period when excluding visual saccades that did not match the auditory saccade range. Only bimodal motor neurons are included in these panels; no bootstrap analysis of these curve fits was performed due to the limited numbers of trials available. All other details are as in Figure 5A.

Nevertheless, we took two additional steps to address this question. First, for the sensory period, we expanded the sampling extent by including some non-saccade trials involving targets near or beyond the limits of the oculomotor range but still within the visual scene (148 neurons, targets: ±30° ±42° and ±60° in addition to original target locations. This corresponds to a range of ±72° in eye-centered coordinates). These trials were included on 15.6±10.4% (mean±SD) of the trials and differed only in that the fixation light stayed on and no saccade was required, which allowed us to investigate the sensory period but not the motor period. Figure 6A illustrates an example neuron, showing peaked tuning for visual stimuli but monotonic sensitivity to sound, and Figure 6B shows that the overall population pattern is very similar to that seen for the more limited target sampling within the oculomotor range in the main data set.

Second, for the motor period, we corrected for any effects that systematic differences in visual vs. auditory saccade accuracy might have introduced to the sampling range. Auditory saccades can show some systematic biases like undershooting or upward shifts [20], [21] A tendency to undershoot auditory targets might cause us to undersample the more eccentric movement amplitudes for sounds, and would bias us towards concluding open-ended coding for sounds. Accordingly, to ensure matched visual and auditory sampling, we limited the *visual* data to visual saccades that spanned the same range of space as the auditory saccades. Visual saccades to a given target that lay more than one standard deviation away from the mean auditory saccade endpoint for that target were excluded from the analysis. Some cells had to be eliminated from the analysis due to too few trials (i.e. if fewer than 20% of the trials were left from the original), and the reduction in number of trials prevented use of the bootstrap analysis. But among those neurons that remained (N = 56 cells), the overall pattern was the same (Figure 6C). If the apparent open-ended response patterns on auditory trials were due to inadequate sampling, then when we make the visual sampling identical to the auditory sampling, the visual code should look open-ended too, but it does not.

### Comparison of monotonicity for visual vs. auditory stimuli

**Figure 7:**
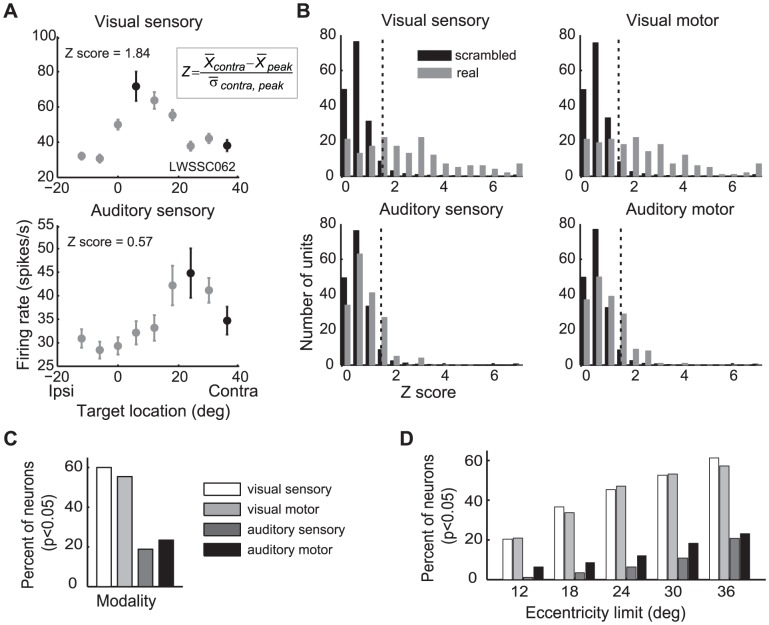
Monotonicity index methods and results. An example neuron showing a drop-off in responses at the most contralateral target positions (sensory responses shown) (A). We compared the responses at the peak location to the responses at the most contralateral location (black dots) and expressed the result as a Z-score (inset). Data for the ipsilateral fixation was used for this analysis. B. The distribution of Z scores for each modality (grey bars), in comparison to the Z scores expected if the relationship between activity and target location is scrambled (Monte-Carlo simulation, black bars). The dotted lines illustrate the 95% confidence threshold; real Z scores to the right of this point are considered to show statistically significant decrements in activity for more peripheral targets (p<0.05) C. The proportion of neurons showing significant non-monotonicity. D. Same as C, but for targets limited to different cut-off points in our sampling range. The disparity between visual and auditory non-monotonicity is present for all cut-offs, and only with a 36 degree cutoff for sound does the level of non-monotonicity reach that seen for a 12 degree cutoff for visual stimuli.

Although the auditory response patterns were generally open-ended, they were not always perfectly monotonic. In some neurons, the responses for the most contralateral target were a little lower than they were for sounds at more intermediate locations (Figure 7A, also see Figure 3A). However, this drop-off was usually small and could have been due simply to variability in neural responses.

To determine how often the drop-off exceeded chance variation, we compared the activity at the most contralateral position with the activity at the best location (Figure 7A and inset). For the neuron shown in Figure 7A, the best location on visual trials (top) was about 6 degrees to the right, and the activity evoked at that location exceeded that for the most contralateral location by about 30 spikes per second. On auditory trials (bottom), the best location was about 24 degrees to the right, exceeding the most contralateral location's response by about 10 spikes per second. To take into consideration the variability in as well as the difference in responsiveness, these values were then converted to a Z score (Figure 7A inset). A large Z score indicates a large drop from the peak activity for more eccentric targets, i.e. a non-monotonic (circumscribed) response pattern. The visual response pattern of the neuron shown in Figure 7A had a Z score of 1.84, whereas the auditory response pattern's Z score was 0.57.

These values mean little on their own, but can be compared to the expected distribution of the Z scores under chance. To calculate this distribution, we performed a Monte-Carlo simulation in which the actual target location was scrambled (Figure 7B). For auditory responses, only about 21% of neurons showed a non-monotonic pattern or Z score that was significantly larger than expected by chance (i.e. was greater than 95% of the scrambled Z scores, dotted vertical lines, p<0.05, grey bars in top panels of Figure 7B). In contrast, almost 60% of visual responses met the criteria (grey bars in bottom panels of Figure 7B). The percentages of neurons exceeding criteria are illustrated in Figure 7C. This discrepancy between the visual and auditory patterns remains even when data for more eccentric locations are excluded, limiting the sampling to smaller ranges (Figure. 7D), again suggesting that sampling range cannot account for the pattern of results.

### A read-out algorithm: a possible way to resolve the discrepancy of coding formats

How is the location of a target extracted from the pattern of SC activity? The SC controls saccadic eye movements, which are generally similar regardless of target modality [22]. Yet SC neurons respond differently depending on target modality, even during the motor period when their activity is most likely to contribute to saccade programming. Thus, the premotor circuitry between the SC and the eye muscles must somehow resolve these differences. Either this downstream circuit must successfully read signals in both formats regardless of modality through a read-out algorithm that is robust to these differences, or there must be a modality-dependent switch that adjusts the SC’s readout depending on the target modality.

**Figure 8:**
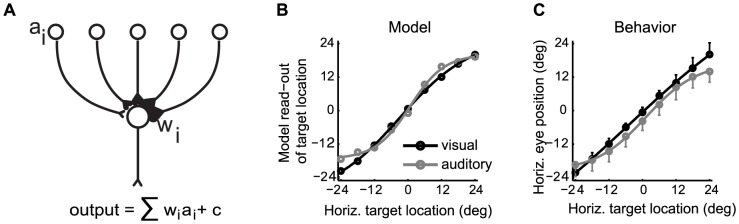
Read-out of SC motor activity for visual and auditory saccades. A read-out model involving graded weights depending on the location of neurons in the SC (A). The weights were fit based on the motor activity on visual trials, combining all fixations and producing an eye-centered estimate of target location. B. Results of the simulation indicate that the model can successfully calculate target location from the input pattern, regardless of modality. C. Behavioral estimates of target location for visual and auditory trials (data from trials during the recording of the neurons). A slight compression of auditory space relative to visual space is seen both for the model (B) and the actual behavior (C).

As proof of concept of the first option, we conducted a simulation to determine if a candidate circuit for reading the SC’s visual signals might be able to read auditory signals as well. We employed a model in which the weighted sum of activity in the SC was calculated [23−25]. The weights were determined from a training set based on the visual motor responses of the neurons in our data set (Figure 8A, see Materials and Methods). The output of the model was then tested using either the visual or auditory motor activity of our neurons as inputs.

The model successfully produced an estimate of visual target location from our recorded SC visual activity that scaled well with target location (Sigmoidal fit, R^2^  =  0.99, Figure 8B, black line). This is to be expected because the visual responses were used to establish the weights. More importantly, the model also performed well when the auditory activity patterns were fed into the model (Sigmoidal fit, R^2^  =  0.99, Figure 8B, grey line). Again, the model’s estimate of target location scaled accurately with the actual auditory target location, and there was no overall offset. Even though we made no attempt to equate the intensity of the auditory stimuli in comparison to the visual stimuli, the model produced a very similar estimate of target location regardless of target modality. A likely reason the model worked is that when calculating a weighted sum, the same answer can be produced either because of the weights (w_i_) – the location of activity for visual trials – or the sum – the amount of activity (a_i_) for auditory trials. For example, an output value of 15 arbitrary units can be produced either because 1 input neuron fires 5 spikes each of which is multiplied by a synaptic weight of 3, or because 5 input neurons with graded weights [1], [2], [3], [4], [5] (an average weight of 3) all fire 1 spike each.

The modality-dependent difference in the model’s output that does exist is subtle, but it is mirrored in the animals’ behavioral performance. The model produces a slight compression of space for sounds compared to visual stimuli (compare the grey and black lines in Figure 8B and note the more sigmoidal shape for auditory targets). A similar compression of space is evident in the monkey’s behavior on auditory trials (Figure 8C). This behavioral pattern may be unavoidable because the SC, by using a read-out algorithm calibrated for visual targets, provides a slight underestimate of the eccentricity of sound locations to the oculomotor premotor circuitry.

The above simulation does not rule out a modality-dependent adjustment to the read-out algorithm, particularly in concert with other potential read out algorithms (see ref. [23]). For example, under vector averaging the level of activity in the SC would be removed through a normalization mechanism – seemingly inconsistent with our findings - but this algorithm could nevertheless produce an accurate SC read-out if there is sufficient heterogeneity in the positions of the near-edges of the auditory movement fields to form a quasi- place code, as has previously been suggested [6]. Such heterogeneity would create a point-image of activity that was larger and centered more caudally in the SC for more eccentric targets than for less eccentric ones. The output of a vector averaging computation would therefore vary proportionately with target eccentricity, but might require an additional modality-dependent adjustment to ensure the resulting movement had the correct gain when the target was auditory.

## Discussion

Similarity of coding of visual and auditory space has long been assumed to be a prerequisite for integrating information from these two sensory systems. While many previous studies have tested the primate SC’s responses to sounds [18], , to our knowledge we are the first to quantitatively characterize the shape of auditory spatial sensitivity for the population of recorded neurons.

Here we have shown that auditory-evoked activity in the SC involves a format different from that of visual-evoked activity in the same population of neurons. This format difference was evident in three different types of analyses: the "point image" of auditory-evoked compared to visually-evoked activity across the population (Figure 4), the curve fitting analysis comparing Gaussian and sigmoidal fits (Figures 5-6 and Figure S1-7), and the monotonicity analysis (Figure 7). These converging lines of evidence suggest that the difference in format is robust to the type of analysis or sampling range used to investigate it.

The predominantly monotonic response patterns for auditory targets occur even in the motor-related activity, which likely is involved in programming saccades to both visual and auditory targets In our sample, non-monotonicity of auditory response functions was slight although not completely absent. If the SC contained an auditory map of space, neurons with the closed field structure indicative of participating in such a map should be *abundant*: their closed fields should tile the oculomotor range and should ensure adequate representation of central locations. This is the case for visually-evoked but not auditory-evoked activity in the SC. 

**Figure 9:**
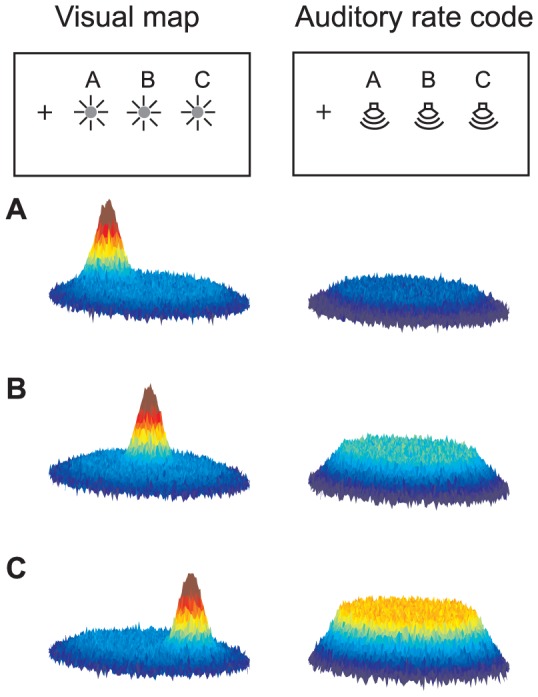
Schematic of the differences in activity evoked in the SC by visual and auditory targets. When a target is visual, a "hill" of activity will be evoked at a location in the SC that corresponds to the visual response field of the neurons. Visual stimuli at different locations would evoke activity at different sites in the SC (A, B, C left panels). In contrast, auditory stimuli at different locations will evoke activity across the SC but with different discharge rates (A, B, C right panels). We note that this schematic does not address the code for the vertical dimension, nor does it consider the possibility that the inflection points of auditory response functions might vary with location in the SC. If the latter is true, then the auditory code would show some topography, with the edge of a broad hill varying with target location.

Although our study focused on the response patterns of individual neurons, the impact of these individual response patterns on the aggregate population response can be visualized as shown schematically in Figure 9. A visual target would evoke a "hill" of activity at a particular location in the SC, corresponding to the bounded visual-sensory and visual-motor response fields of neurons at that site (left column). Different visual locations would evoke activity at different sites. In contrast, auditory targets at the same set of locations would evoke activity throughout the SC but at a level that varied with sound location.

The disparities we observed between the visual and auditory codes in the primate SC illustrate that bimodal populations of neurons can use different ways of representing different sensory signals. Such differences may account for subtle modality-dependent differences in the behavioral responses guided by such populations.

Exactly how the disparities are resolved as signals progress from the SC to the muscles controlling eye movements is not yet clear. Our modeling suggests that it is in principle possible for the circuit that "reads" the SC to produce a similar answer for a similar target location despite modality-dependent differences in the activity evoked. Further experimental work will be needed to determine if this in fact what happens or if the circuitry intervening between the SC and the extraocular muscles interprets SC activity differently depending on target modality.

Receptive fields that do not fully close on the eccentric side have been reported in the superior colliculus before. Such response patterns have been most extensively characterized in the monkey SC by Munoz and Wurtz [30], who reported that about a third of SC neurons with motor-related activity to visual targets did not exhibit a clear outer boundary to their movement fields. Some of the examples shown in that study exhibited monotonic responses to target eccentricity whereas others showed a clear peak response to nearer targets and considerably weaker responses to more eccentric ones. In our study, we emphasized monotonicity as the key factor, and we report that monotonicity is substantially more prevalent when saccades are evoked by sounds than by visual stimuli.

Closed-field receptive fields do not appear to be required for coding locations in frontal, horizontal auditory space. Neurons with open-ended spatial receptive field have been found in many mammalian brain areas including the superior olive [31−33], inferior colliculus [15], , medial geniculate body [38], [39], and auditory cortex [12], [17], . The majority of spatially-selective neurons in these studies appear be most responsive to lateral locations [12], [17], [34−36], [41], . Recent studies using imaging suggest that sound location coding in human auditory cortex is largely similar to these animal studies, and involves an overall monotonic pattern of azimuthal sensitivity [13].

The emerging picture from a variety of species and brain areas suggests that spatial location may often be encoded via “meters” rather than maps. In a meter, the level of activity in a population of broadly-responsive neurons can provide an indication of the location of sounds [13], [14], , an idea that originated with von Bekesy [49]. Of course, maps of neurons with circumscribed receptive fields could be computed from these meters [16], and this process may occur in some species. In the primate, such maps remain to be discovered.

Finally, it should be noted that here we have only varied the *horizontal* component of sound location. How SC neurons signal the vertical component of sound location is unknown. Vertical information derives exclusively from position-depending differences in the frequency filtering properties of the external ear, known as spectral cues. Unlike interaural timing or level differences which vary monotonically with sound azimuth, the relationship between spectral cues and sound elevation is complex. The SC and other auditory-responsive structures may therefore use a quite different method for encoding this dimension of the auditory scene.

## Materials and Methods

### Ethics statement

Two rhesus monkeys (*Macacca mulatta*, one male and one female, ages 4−9) participated in the studies. The animal experimental protocols were approved by the Institutional Review Board and the Institutional Animal Care and Use Committee of Duke University, respectively. Animal procedures were conducted in accordance with the principles of laboratory animal care of the National Institutes of Health (publication 86−23, revised 1996) and involved standard operative and post-operative care including the use of anesthetics and analgesics for all surgical procedures.

Specifically, prior to surgery, animals were pre-anesthetized with ketamine (I.M., 5−20 mg/kg) or ketamine/dexdomator (I.M., 3.0 mg/kg ketamine and 0.075−0.15 mg/kg dexdomitor) and were maintained under general anesthesia with isoflourane (inhalant, 0.5−3.0%). Systemic anti-inflammatory medications (dexamethasone, flunixin, or keterolac) were given as indicated by veterinarian. After surgery, animals were treated with burprenorphine analgesic (I.M., 0.01−0.02 mg/kg doses) for three days.

Animals were housed in a standard macaque colony room in accordance with NIH guidelines on the care and use of animals. Specifically, the animals were housed in Primate Products Apartment Modules (91 cm*104 cm*91 cm), including pair or group housing when compatible partner monkeys were available. Pair and group housed animals exhibited species-typical prosocial behavior such as grooming. Animals also had frequent access to Primate Products Activity Modules (91 cm*104 cm*183 cm), allowing for more exercise including a greater range of vertical motion. All animals had visual and auditory interactions with conspecifics in the room (∼10 animals). Animals were enriched in accordance with the recommendations of the USDA Environmental Enhancement for Nonhuman Primates (1999), and the National Research Council’s Psychological Well-Being of Nonhuman Primates (1998), and the enrichment protocol was approved by the IACUC. Specifically, the animals had access to a variety of toys and puzzles (e.g. Bioserv dumbbells (K3223), Kong toys (K1000), Monkey Shine Mirrors (K3150), Otto Environmental foraging balls (model 300400) and numerous other toys and puzzles). Material from plants such as Bamboo and Manzanita was also placed in the cage to give the animals additional things to climb on and interact with. The temperature in the animal facilities was 20−25 degrees C and the colony room was kept on a 12hr/12hr light/dark cycle. The animals had approximately an hour of audiovisual contact with at least two (and often several) humans per day. The animals’ diets consisted of monkey food (LabDiet 5038 or LabDiet 5045) delivered twice a day, plus daily supplementary foods such as bananas, carrots, mango, pecan nuts, dried prunes, or other treats (typically acquired from local supermarkets or online vendors) to add variety to the animals’ diets. No animals were sacrificed during this study - at the time of the submission of this manuscript both animals that participated in this study are in good general health.

### Animal preparation

The subjects underwent sterile surgery for the implantation of a head post holder, eye coil and recording chamber [50], [51]. After behavioral training, a recording cylinder was implanted over the left (Monkey W-male) and right (Monkey P-female) SC using stereotaxic techniques. The location of the cylinder was verified with MRI scans at the Duke Center for Advanced Magnetic Resonance Development.

### Experimental setup and stimuli

All experimental and behavioral training sessions were conducted in a dimly illuminated sound-attenuation room (IAC, single-walled) lined on the inside with sound-absorbing foam (Sonex PaintedOne). Stimulus presentation and data collection were controlled though Gramakln 2.0 software (Ryklin Software, developed from the laboratory of Dr. Paul Glimcher). Eye position was sampled at 500 Hz. EyeMove (written by Kathy Pearson, from the laboratory of Dr. David Sparks) was used to analyze the eye position traces. Velocity criteria to detect saccade were 20 degrees/s for both saccade onset and offset. All subsequent analysis was performed in Matlab 7.1 (Mathworks software).

Sensory targets were presented from a stimulus array which was 58 inches in front of the monkey. The array contained nine speakers (Audax, Model TXO25V2) with a light-emitting diode (LED) attached to each speaker’s face. The speakers were placed from 24° left to 24° right of the monkey in 6° increments at an elevation of 0° (Figure 2A), with additional speaker-LED assemblies positioned at ± 30°, 42° and 60° for the non-saccade task described elsewhere. Fixation LEDs were located 12° left, 0° and 12° right, and were positioned above or below the row of LED-speaker assemblies such that the latter were within the receptive/movement field of the neuron under the study.). Auditory targets were band-pass white noise burst (500 Hz to 18 kHz; rise time of 10 ms) at 50dB ± 2dB SPL. The luminance of each LED was ∼26.4cd/m2.

### Behavioral Task

The monkey performed an overlap saccade task to auditory and visual targets, in which all conditions were randomly interleaved (Figure 2B). After fixating a fixation target for 900 – 1,200 ms, a visual or auditory target was presented. After a delay of 600−900 ms, the fixation light was extinguished and the monkey had 500ms to shift its gaze to the location of the target to receive a liquid reward. For 148 neurons, an additional non-saccade task (Figure 2C) was interleaved on average 15.6% of trials. This task was similar to the overlap saccade task except that the target locations were near or beyond the oculomotor range, the fixation light stayed on and no saccade was required.

### Recording Procedure and Strategy

At the start of each recording sessions, a stainless-steel guide tube was manually advanced through the dura. Next, the monkeys performed the overlap saccade task while a tungsten electrode (FHC, impedance between 2 and 4.5 MΩ at 1 kHz) was extended further into the brain with an oil hydraulic pulse micropositioner (Narishige-group.com). Extra-cellular neural signals were amplified and action potentials from single neurons were isolated using a PLEXON system (Sort Client software, PLEXON.INC). The time of occurrence of each action potential was stored for off-line analysis.

When a neuron was isolated, we qualitatively determined the elevation of the receptive or movement field while monkeys performed the overlap task. The elevation of the fixation was chosen near that preferred elevation of the neuron on that session. The target modalities (auditory VS visual), the locations of fixation and the locations of targets were randomly varied on a trial by trial basis. Data were collected as long as the neuron was well isolated and the monkey performed the task. On average, we collected 11.16 ± 5.41 (mean °± SD) successful trials per task condition (fixation location x target location x target modality).

### Data Analysis

We analyzed neural activity by counting action potentials during several time periods. The baseline period was the 500 ms before target onset, and the sensory target period was the 500 ms period after target onset (Figure 2B). The motor-related activity period was synchronized with the saccade, beginning 20ms before saccade onset and ending 20ms before saccade offset (latencies chosen based on the minimum latency of stimulation-evoked movements in the SC [52−55]). The sensory and motor time windows did not overlap as the monkeys were cued to withhold the saccade to the target by the continued presence of the fixation light for at least 600−900 ms after target onset. Counts of action potentials were converted to discharge rates to account for the differences in duration of these different time periods. The sensory period activity was analyzed with respect to the horizontal component of target location, and the motor period activity was analyzed with respect to the horizontal component of the movement to that target location, thus taking into account any variations in behavioral response and concomitant neural variations. Neural responses were converted to spike rates.

### Curve fitting

Sigmoidal and Gaussian curve fitting was accomplished as follows. Both curves had the same number of free parameters (i.e. 4). The Gaussian equation was
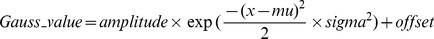



And the sigmoidal equation was
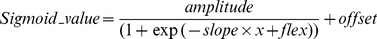



The sigmoidal and Gaussian curve fitting was accomplished using Matlab and the “lsqnonlin” function, which involves an iterative search to minimize the least squares error of the function. We found the optimal curve fits using a variety of initial starting conditions. Each curve fit was also visually inspected for adequacy.

### Neuronal population

The intermediate and deep layers of the SC provided the bulk of the recorded neurons. Neurons were included for the relevant analysis if they responded significantly during the sensory or motor-related periods on visual or auditory trials compared to baseline (two-tailed paired t test, p<0.05). For this t-test, all target locations were pooled together because this proved to be the most inclusive criterion. The majority of neurons showed significant responses to both visual and auditory targets during the saccade-related bursts (Table 1). There were very few neurons that showed saccade-related bursts for only one modality (n = 11, 7% out of 162 visual neurons, n = 8, 5% out of 159 auditory neurons), indicating that the overwhelming majority of neurons that exhibited saccade-related bursts were recruited regardless of target modality. A smaller majority of neurons were bimodal during the sensory period (n = 113, 63% out of 180 neurons, visual only: n = 46, 29% out of 159 visual neurons and auditory only: n = 9, 7% out of 122 auditory neurons).

### Microstimulation

To assess the locations of the recording sites with respect to the SC’s motor map, we microstimulated using standard techniques after recording at 19 sites in monkey P. These sites were distributed in 5 out of the 6 guide tube locations which we used for recording from monkey P. During each microstimulation session, the monkey performed a task involving fixating one of three initial eye positions (−12, 0 and 12 degree from the center). The vertical position of the fixation LEDs was the same as in the immediately prior recording session. After fixating for 150ms, the fixation LED was turned off and a stimulation train was applied for 150ms. To allow the subject to earn a reward unconnected to any evoked saccade, a visual saccade target was presented 300ms after the stimulation (20° above fixation and −12, 0 and 12° to the left or right). For comparison purposes, catch trials, identical but without stimulation, were presented 20% of the time.

### Simulation of circumscribed and open-ended response functions

The circumscribed receptive fields shown in Figure 5B were simulated with Gaussian functions varying in peak location, tuning width and height. Open-ended monotonic response patterns were simulated with sigmoidal functions varying in inflection point, slope, and height. The responses on individual trials were simulated by adding noise drawn from a normal distribution. The resulting activity patterns, whether Gaussian or sigmoidal in origin, were then in turn fit by both Gaussian and sigmoidal functions.

### Read-out model

Each neuron was included in the model twice, as originally sampled and mirror flipped as if it were recorded in the SC on the opposite side. A training set to establish weights was created from *only the visual* motor responses. The mean and standard deviation of the responses to visual targets at each target location was calculated, and then draws were made from a normal distribution with those parameters. The weights were fit using a least-squares regression equation with each neuron providing a term to the equation: 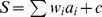



where S is the amplitude of the saccade, and w_i_ and a_i_ are the synaptic weight and motor activity level of the *ith* neuron and c is a constant term. The model was then tested by plugging in the mean visual motor responses and the mean auditory motor responses. We used the motor period rather than the sensory period because the reference frame of visual and auditory signals is more similar (i.e. auditory signals are more fully eye-centered and thus better matched to the visual reference frame) during this trial epoch [18].

## Supporting Information

Figure S1 **Gaussian – sigmoid R^2^ plots** **with** **bimodal neurons.** This figure corresponds to Figure 5A in the main paper except that the sample is limited to neurons with sensory or motor activity for both visual and auditory targets.
(EPS)

Figure S2 **Gaussian - sigmoid R^2^ plots colored by the eccentricity.** We re-plotted the R^2^ values from Figure 5A of the main paper with using color to indicate the eccentricity of the peak of the Gaussian (for visual activity) or inflection point of the sigmoid (for auditory activity), similar to the simulation in figure 5B. For visual responses, sigmoidal and Gaussian fits were more comparable if the Gaussian peak was more eccentric (warmer colors for the data points near the line of slope 1). This was not seen for auditory activity.
(EPS)

Figure S3 **Saccades evoked by microstimulation at 19 representative sites in the SC.** Average saccade vectors evoked by microstimulation at the central fixation position (A). B. Distribution of the horizontal component of the microstimulation evoked saccades. Triangle indicates the mean value. C. Gaussian-sigmoid R^2^ plots for the neurons recorded at these sites. The pattern is very similar to that of the main data set (Figure 5A bottom panels).
(EPS)

Figure S4 **Results using the correlation coefficient, R, rather than the coefficient of determination, R** ***^2^*** **.** These data represent the square root of the values shown in Figure 5A. This figure provides a point of direct comparison with our previous studies for which we plotted the correlation coefficient [12], [15].
(EPS)

Figure S5 **Comparison of the relative magnitudes of the Gaussian and sigmoidal R^2^ values.** The histogram in each panel involve the subtraction of the sigmoidal R^2^ from the Gaussian R^2^ values illustrated in Figures 5A, 6B, and 6C for the data sets shown. The visual and auditory distributions in all four panels differ significantly from one another (t-test, p<0.05).
(EPS)

Figure S6 **Relationship between response amplitude and the Gaussian vs. sigmoidal R^2^ comparison.** In bimodal neurons, average visual sensory responses tended to be slightly stronger than average auditory sensory responses (data points below the line of slope one) (A, see Table 1). 113 neurons were included and the data for each fixation position were treated separately, yielding N = 339 condition data points (grey dots). Responses were averaged across target locations. To determine if differences in response amplitude affected the curve-fitting analysis, we selected a subset of cases where this difference was minimal, i.e for which the difference in responsiveness for the two modalities was fewer than 10 spikes/s (Number of conditions = 113, red dots). B-C. The Gaussian-sigmoid R^2^ plots for this subset exhibited a pattern very similar to that of the main data set (Figure 5A Top panels). D-F. Same as A-C but for the motor period (Number of bimodal neurons = 151; see Table1; number of conditions  =  453, grey dots). A slightly larger criterion of 15 spikes/s was chosen to more closely match the number of conditions included (N  =  129, red dots). These results suggested that the difference in response amplitude is not likely to account for the difference between the visual and auditory curve fitting results.
(EPS)

Figure S7 **Relationship between response variability and the Gaussian vs. sigmoidal R^2^ comparison.** Similar to Figure S6 but assessing the effects of response variability instead of amplitude. Criteria for inclusion for analysis was a variance difference of fewer than 2 spikes^2^/sec^2^ for the sensory period (N = 111, red dots; A-C) and fewer than 6 spikes^2^/sec^2^ ( for the motor period (N = 101, red dots; D-F). Note that the scale of variance for the motor activity is triple that for sensory activity, and the larger criterion for the motor period permits a rough match between the numbers of conditions included in the subset for both analyses. Overall, the pattern is again very similar to that of the main data set (Figure 5A Bottom panels) suggesting that differences in response variability are not likely to account for the difference between the visual and auditory curve fitting results.
(EPS)
